# Sustainability and resilience of health system financing in Egypt: a mixed qualitative methods study for evidence-informed policymaking

**DOI:** 10.1016/j.hpopen.2026.100194

**Published:** 2026-10-28

**Authors:** Ranin Soliman, Dan Gocke, Elias Mossialos, Hesham Dinana

**Affiliations:** a School of Life and Medical Sciences, University of Hertfordshire Hosted by Global Academic Foundation, Egypt; b Evidence and Data to Policy Unit, Division of Science, Information and Dissemination, WHO Regional Office for the Eastern Mediterranean (WHO EMRO), Egypt; c Department of Health Policy, London School of Economics and Political Science, UK; d Department of Journalism and Mass Communication, American University in Egypt, Egypt

**Keywords:** Health financing, Sustainability, Resilience, Health policy, Evidence-based policymaking, Egypt

## Abstract

**Introduction:**

The Egyptian health care system is undergoing significant transformation towards universal health coverage, and it is, therefore, necessary to evaluate the sustainability and resilience of the health system financing.

**Aim:**

Our study aims to analyze the sustainability and resilience of health system financing in Egypt and provide evidence-based recommendations for evidence-informed policymaking. This was achieved through: (1) studying the current financing mechanisms of Egypt's health system; (2) determining how sustainable are these financing mechanisms given current trends; and (3) evaluating how resilient was the Egyptian health system financing during the COVID-19 pandemic.

**Methods:**

The study was conducted from July 2023 to December 2023 over three stages: (1) literature review, (2) semi-structured interviews with 16 key stakeholders, and (3) focus groups with 12 experts. We conducted qualitative data analysis using a framework analysis approach, with findings triangulated across the three data sources. Narrative synthesis was used to summarize the main findings.

**Findings:**

The health system in Egypt is currently financed through the old health insurance system, the newly implemented universal health insurance (UHI) system, private insurance system, and out-of-pocket financing. Financial indicators showed improved sustainability of health financing. Between 2010 and 2020, Egypt's health spending as a percentage of GDP increased, while out-of-pocket spending as a percentage of current health expenditures decreased. However, significant structural challenges persist, including a heavily regressive financing structure and coverage gaps for vulnerable populations. The UHI program ensures sustainability of health financing for the whole population in the longer term. Key insights demonstrated the resilience of the Egyptian health system financing during the COVID-19 pandemic, where supplementary public funds were released to cover the additional pandemic-related expenditures. **Conclusion:** This study provides valuable insights and evidence-based recommendations for policymaking to promote the sustainability and resilience of health system financing in Egypt. Recommendations are organized by health system sustainability and resilience domains and explicitly linked to policy implications to support evidence-informed policymaking.

## Highlights

• The study combines mixed methods including literature review, interviews and focus groups.
• Egypt's health expenditure profile reflects initial improved sustainability in 2005–2020.
• Health financing is largely based on out-of-pocket payments and is mainly regressive.
• During the COVD–19 pandemic, health financing showed resilience using public funds.
• Egypt's health system is moving towards value-based healthcare financing.

## 1 Introduction

The Egyptian healthcare system is currently undergoing great transformations towards universal health coverage (UHC), as part of ‘Transforming Egypt's Healthcare System’ project led by the World Bank. [1] This is aligned with Egypt's vision 2030 and achieving the health-related sustainable development goals (SDGs). [2] The World Health Organization (WHO) works with the Ministry of Health and Population (MoHP) and other national health authorities to support health system financing in the pursuit of UHC to find options to improve the health financing system. [3]

Since Egypt is a lower-middle income country according to the World Bank's classification, [4] and given the large population of Egypt (114,535,772 million in 2023) with a yearly growth rate of 1.7% in 2023. [5] It is necessary to evaluate the sustainability and resilience of the health system financing to ensure the delivery of high-quality equitable health services, maintain financial protection, and resilience to adapt to crises. However, limited published data exist about the sustainability and resilience of the healthcare system in Egypt, including the financing scheme of the healthcare system in Egypt, the mechanisms to support these efforts, and future recommendations to better maintain the sustainability of health system financing within Egypt's vision 2030 and its related SDGs. A systematic literature review conducted by Fasseeh et al. (2022) summarized findings from previously published studies about healthcare financing mechanisms in Egypt, nevertheless, none of these studies addressed or evaluated the sustainability and resilience of the health system financing. [6] Besides, none of the conducted studies generated qualitative evidence based on interviews with key stakeholders or focus groups with the experts, [6] highlighting a gap in the existing literature that evaluates the health system financing in Egypt based on quantitative indicators and qualitative insights based on experts' opinions.

The Partnership for Health System Sustainability and Resilience (PHSSR) framework model proposes a methodology for evaluating the sustainability of health systems and their resilience to crises based on five areas: health system management; financing; medical personnel; medicines and technologies; organization and provision of medical care. [7] For the purposes of this study, sustainability refers to the long-term capacity of the health financing system to mobilize and allocate adequate resources to meet population health needs without creating financial hardship. [7] Resilience refers to the ability of the health financing system to absorb, adapt, and recover from shocks such as economic crises or pandemics. [7] These two dimensions are conceptually distinct yet interrelated, where a sustainable system must be resilient to endure shocks, while a resilient system requires sustainable financing to maintain functioning during and after crises. [7]

Our study aimed to analyze the sustainability and resilience of health system financing in Egypt following the PHSSR framework and provide evidence-based recommendations for informed policymaking. This study was guided by the following three research questions: (1) What are the current mechanisms of health system financing in Egypt, and how do they interact to support universal health coverage; (2) To what extent is Egypt's health system financing sustainable, based on trends in health expenditure indicators between 2010 and 2020; and (3) How resilient was the Egyptian health system financing during the COVID-19 pandemic, and what mechanisms were activated to maintain financing continuity.

## 2 Methods

### 2.1 Study design

We adopted a framework model developed by the London School of Economics (LSE) research group in the context of the Partnership for Health System Sustainability and Resilience (PHSSR). [8] The LSE PHSSR framework consists of seven core domains: health system governance, health system financing, health system workforce, medicines and technology, health service delivery, population health, and environmental sustainability. [8] This study focused on the health system financing domain and was conducted from July 2023 to December 2023 using three data sources for data collection: (1) literature review, (2) semi-structured interviews with key stakeholders; and (3) focus groups with the experts.

### 2.2 Data source 1: literature review

The literature review was conducted on PubMed and Google Scholar on 20th August 2023, using the keywords: “health* financing” OR “health* expenditure*” OR “health* economics” AND “Egypt”. Additionally, we conducted a grey literature review and searched website of the MoHP and other national authorities in Egypt to gather information about health system financing in Egypt. Inclusion criteria for the review encompassed peer-reviewed articles and official reports published between 2000 and 2023, in English or Arabic, focusing on health financing in Egypt. Data on health expenditure indicators were extracted from the Egypt National Health Accounts 2019/2020 report, [9] and the WHO Global Health Expenditure Database. [10]

### 2.3 Data source 2: stakeholder interviews

Semi-structured interviews were conducted with 16 key stakeholders purposively selected to represent diverse expertise across health financing, health economics, and health policy in Egypt. Participants were identified through a combination of the PHSSR expert network, purposive sampling and snowball sampling. Eligibility criteria included: (i) at least five years of professional experience in health financing or health policy in Egypt, and (ii) familiarity with either the old health insurance system or the new UHI system. Data saturation was assessed iteratively; no new themes emerged after the 14th interview, and two additional interviews were conducted to confirm data saturation. All interviews were conducted in Arabic or English based on participant preference, audio-recorded with consent, and professionally transcribed verbatim.

The interviews covered the following four areas health system financing based on the PHSSR framework developed by the LSE research team: (1) budgeting and crisis preparedness; (2) revenue generation and spending; (3) coverage and resource allocation; (4) key trends in health system financing. An interview guide based on the PHSSR health system financing areas is presented in **Supplementary Table S1**.

### 2.4 Data source 3: expert focus groups

Focus groups were conducted with 12 relevant key experts through round table discussions in a one-day event organized on 14th November 2023 in Cairo, Egypt. Participants included health economists, public health specialists, policy analysts, and representatives from the Ministry of Health and Population. Focus group sessions were moderated by two members of the research team using a semi-structured guide aligned with the PHSSR financing domain [**Supplementary Table S1]**. Sessions were recorded and transcribed verbatim.

### 2.5 Data analysis and synthesis

Qualitative data analysis was conducted to summarize the main findings obtained through the literature review, interviews, and the focus group discussions. We conducted thematic analysis to identify the four PHSSR financing sub-domains (themes): (i) revenue generation and expenditures, (ii) coverage and resource allocation, (iii) provider payment mechanisms, and (iv) crisis preparedness and resilience. We followed the six-phase thematic analysis framework developed by Braun and Clark (2006), [11] which includes the following steps: (1) familiarization with the data; (2) generating initial codes; (3) searching for themes; (4) reviewing themes; (5) defining and naming themes; and (6) writing the final themes. [11] Deductive thematic data analysis was performed to summarize the main findings obtained from the literature review, interviews, and the focus group discussions into the four PHSSR sub-domains. Three members of the research team independently coded a sample of 20% of the interview transcripts; inter-rater reliability was discussed and discrepancies were resolved by consensus. Triangulation was achieved by systematically comparing findings from the three data sources (literature, interviews, focus groups): convergent findings were treated as confirmed; divergent findings were flagged and explored for contextual explanations.

Narrative synthesis was done through combing the information obtained from the three data sources drawing on the latest publicly available information, relevant stakeholders' and experts' opinions. We also made tabulation of the main findings, and the strengths and weaknesses regarding the sustainability and resilience of health system financing in Egypt, as a main outcome measure of this research work. We also presented a set of recommendations aimed at strengthening the sustainability and resilience of financing the health system in Egypt.

### 2.6 Ethical considerations

This study was approved by the institutional review board (IRB) at the American University in Cairo, Egypt (CASE #2024–2025-003) to conduct the interviews with key stakeholders and the focus groups with experts. Confidentiality was maintained by anonymizing all interview and focus group data; no identifying information is reported in this manuscript. Participants were made aware of their right to withdraw at any time without consequence.

## 3 Results

An overview of the identified strengths and limitations regarding the sustainability and resilience of health system financing in Egypt, mapped to each data source (literature review, semi-structured interviews, and focus group discussions) is presented in Table 1. Findings were organized in accordance with the three research questions guiding this study about (1) health system financing mechanisms; (2) sustainability of health system financing; and (3) resilience of health system financing, and sub-categorized by the PHSSR sub-domains under each of the health system sustainability and resilience sections.

**Table 1:** Overview of the identified strengths and limitations regarding the sustainability and resilience of health system financing in Egypt, mapped to each data source.

	Sustainability of health system financing	Resilience of health system financing
Strengths	• Health spending as a percentage of GDP increased between 2010 and 2020, reflecting potential improvement in sustainability of health financing (Egypt National Health Accounts 2019/2020 report. WHO/EMRO. 2023). • Out-of-pocket (OOP) spending as a percentage of current health expenditure decreased from 62.6% in 2010 to 59.3% in 2020 (Egypt National Health Accounts 2019/2020 report. WHO/EMRO. 2023). • The new Universal Health Insurance program has the potential to enhance sustainability of health financing as it is predominantly contribution-based and is expected to show an initial large surplus in revenues (Egypt UHI project, 2020; focus group discussions). • Payments to healthcare providers are based on level and type of care delivery. This currently sustains the health system in the short term (Stakeholders' interviews)	• During the COVID-19 pandemic, supplementary public funds were released to cover the additional pandemic-related expenditures, maintain resilience of health system financing during the pandemic (Essam El-Din, 2021; Stakeholders' interviews) • A new bill was approved in 2021 to establish a medical emergency fund to combat emergency medical expenses in Egypt aiming to promote the resilience of the health system financing (Essam El-Din, 2021).
Weaknesses	• The health system in Egypt is largely financed by OOP payments, so the health financing is mainly regressive (Ahmed et al., 2020). • The burden of health financing falls more on the most vulnerable populations due to increased OOP spending, such as the poor, self-employed, rural women and young people aged 20–24 years old, urging a need to achieve more equitable financing (Selwaness, et al. 2019; focus group discussions). • No financial efficiency and health outcomes exist, making it difficult to introduce value-bad payment models that link healthcare spending to population health outcomes (Health Affairs, 2022; Stakeholders' interviews). • The old taxation-based HIO system suffers from financial deficits, affecting the quality and quantity of public health services (Stakeholders' interviews) There are no mechanisms or risk equalization measures to allocate funds based on population needs or disease burden based on real-world evidence from local context (Stakeholders' interviews)	• There are no forecasts about risk of exposure to outbreaks, epidemics, or pandemics limiting the resilience of the health system to cope with any future health shocks (Stakeholders' interviews)

**Notes: GDP:** Gross Domestic Product; **OOP:** Out-of-pocket spending; **HIO:** Health Insurance Organizations.

Sources:

WHO. Egypt National Health Accounts. Establishing an expenditure baseline to support Egypt’s health care reform 2019/2020. Cairo: WHO Regional Office for the Eastern Mediterranean; 2023.

Ahmed, Y., Ramadan, R. and Sakr, M.F. Equity of health-care financing: a progressivity analysis for Egypt. *Journal of Humanities and Applied Social Sciences*. 2020;(1).pp.3-24.

The Arab Republic of Egypt Presidency. National Projects. Universal Health Insurance Project. 2020.

Selwaness I and Ehab M. Social protection and vulnerability in Egypt: a gendered analysis. Working Paper No. 1363. Economic Research Forum. AUC. October 2019.

Health Affairs. Value-Based Payment as A Tool to Address Excess US Health Spending. Research Brief. 2022.

Essam El-Din, G. Egypt's parliament approves bill establishing a fund to spend on medical emergency services. 2021. Ahram Online.

### 3.1 Health system financing mechanisms in Egypt

The health system in Egypt is currently financed through four main mechanisms: (1) the old health insurance organization (HIO), (2) the newly implemented universal health insurance (UHI) system, (3) private insurance, and (4) out-of-pocket (OOP) payments. Fig. 1 presents the proportion and trends in sources of health expenditure between 2000 and 2021, illustrating the dominant and persistent role of OOP payments throughout this period, based on findings from the Egypt National Health Accounts 2019/2020 published by the WHO/EMRO (2022). [9]

**Fig. 1:**
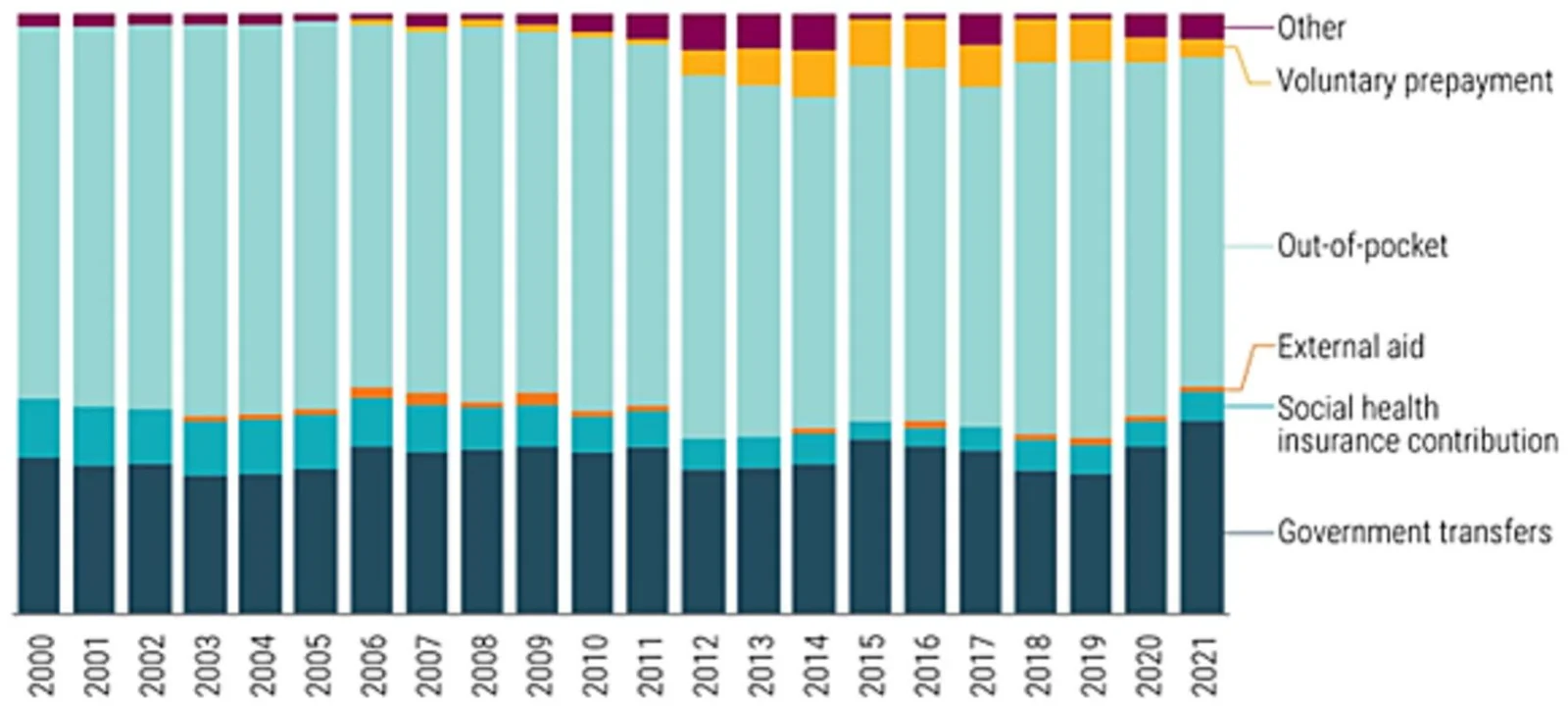
Proportion and trends of sources of health expenditure (percentages), 2000–2021.

As shown in Fig. 1, OOP payments have consistently constituted the largest share of current health expenditure, ranging from approximately 60–65% throughout the 2000–2021 period, with a gradual declining trend from 2015 onwards. Government transfers represent the second largest source, followed by social health insurance contributions which have shown a modest increasing trend. This financing structure reflects a regressive system in which financial burden falls disproportionately on lower-income households.

### 3.2 Sustainability of Health system financing in Egypt

#### 3.2.1 Revenue generation and expenditures

Historically, the Egyptian healthcare system has faced challenges due to limited public investment in the health sector and high out-of-pocket (OOP) expenditures. However, the approval of the new Constitution in January 2014 nearly doubled government expenditure on healthcare from 1.5% to 3% of gross domestic product (GDP), leading to significant improvements in health system financing towards universal health coverage (UHC) in Egypt.

In 2023, the Ministry of Health and the WHO Egypt Country Office released the National Health Accounts (NHA) 2019/2020 report, providing an expenditure baseline to support Egypt's health system financing reform. 9., 10. The trend in health spending per capita increased over time from USD 71.04 in 2000 to USD 173.52 in 2015, and then slightly decreased to USD 150.91 in 2020, (**Supplementary Fig. S1)**. Health spending as a percentage of GDP increased from 4.15% in 2010 to 4.36% in 2020, reflecting improved sustainability of health financing, with government health spending currently in the process of meeting the 2014 Constitution's ambition to reach or exceed 3% of GDP. 9., 10.

The key statistics of Egypt's health expenditure profile between 2005 and 2020 reflect improved sustainability in most health system financing indicators, 8., 9. as shown in Table 2. Health spending per capita increased from USD 107 to USD 174 between 2010 and 2015 and then decreased to USD 151 in 2020. 9., 10. It is important to note that this decrease reflects the 2016 devaluation of the Egyptian Pound (EGP) relative to the US dollar rather than an actual reduction in health spending in local currency terms. [12] Therefore, even if spending per capita increased in terms of EGP, it would show a decrease when expressed in USD due to the change in exchange rate. This is a significant data limitation that should be interpreted with caution when using USD-denominated indicators throughout this analysis. All spending figures in Table 2 are inflation-adjusted using the consumer price index from the Central Bank of Egypt. 9., 10.

**Table 2:** Key statistics of Egypt's health expenditure profile, 2010–2021.

	Year 2010	Year 2015	Year 2020	Year 2021
Health spending, $US per capita (CHE)*	$107	$174	$151	$180
Government health spending as a percentage of CHE	32.9%	31.2%	31.9%	37.7%
Out-of-pocket (OOP) spending as a percentage of CHE	62.6%	59.5%	59.3%	54.9%
Priority to health (GGHE % GGE)	4.4%	5.1%	5.2%	6.8%
Gross Domestic Product (GDP) per capita ($US)	$2586	$3428	$3457	$3898

**Notes: CHE =** Current Health Expenditure; **GGHE =** General Government Health Expenditure; **GGE:** General Government Expenditure. * Health spending per capita adjusted for inflation using consumer price index from the Central Bank of Egypt. **Source:** WHO Global Health Expenditure Database (2024).

The OOP spending as a percentage of current health expenditure (CHE) decreased from 62.6% in 2010 to 59.3% in 2020, as shown in Table 2 9., 10. Nevertheless, despite this modest improvement, OOP spending remains above 59%, indicating that health financing in Egypt is predominantly regressive. A study by Ahmed et al. (2020) showed an overall Kakwani index of −0.079, with regressive effects from OOP payments, earmarked cigarette taxes, and direct taxes, and mild progressive effects from private health insurance and indirect taxes. [13] The rate of OOP health expenditure as a percentage of household expenditure is 9.4%, 9., 10. approaching the World Bank indicator of catastrophic healthcare expenditure, defined as, ‘OOP spending exceeding 10% of household expenditure’. [14] This was calculated by dividing OOP spending per capita $89.51 (USD 2020) based on World Bank data, by annual household expenditure per capita $950.771 (USD 2020). [14] This highlights the continuing need for health financing reform to achieve more equitable financing and reduce the OOP burden, as the burden of health financing particularly falls on the vulnerable populations. [13]

The implementation of the new UHI system will ensure financial protection and health equity for all the population regardless of their ability to pay or other socioeconomic variations, as the financing scheme is a hybrid of contribution payments, social health insurance (tax-based), and earmarked revenues (including cigarette taxes, car tolls, car licensing). [15] Most of the stakeholders in the focus group discussions also agreed that the currently ongoing healthcare transformations towards universal health coverage may help contribute to more revenue generation towards better health system sustainability. This is also in alignment with the insights gained from experts' interviews, where one interviewee mentioned that “*the main source of revenue for the new UHI financing scheme is predominantly contribution-based which is expected to show an initial large surplus in revenues, in contrast to the old health insurance system that is mostly taxation-based*.” Stakeholders consistently highlighted the structural challenge of the old HIO's financial deficits, with one interviewee noting: *“The old system was never adequately funded — providers managed deficits through cross-subsidization, not through a functioning reimbursement model*.” This reinforces the quantitative evidence of a system under fiscal strain despite headline improvements.

Addressing the PHSSR sub-domain of revenue generation and expenditures and the related guiding questions on bailing out public providers [detailed in **Supplementary Table S1**], one expert interviewee in the public health insurance sector stated, “*Over the last ten years, there have been no bailouts of public providers or social insurance funds that ran into deficits, where the public hospitals could either manage these deficits based on the generated revenues from service surplus or through government transfers*.” Another key factor that may affect the sustainability of health system financing is the trend in population demographics. Based on published literature, there is an increasing trend in the aging population (above 60 years old) in Egypt. 16., 17., 18. Nevertheless, the country is not suffering from a narrowing tax base due to the aging population, as around 40% of the population is under 18 years old, as highlighted by a health economics expert during the semi-structured interviews, explaining, “*As of now, the younger population less than 18 years old adds to the tax burden along with the elderly population. However, in the long run, this large young population will enter the workforce and improve the dependency ratio*.”

#### 3.2.2 Coverage and resource allocation

The Public Health Insurance law was approved by the Egyptian parliament in 2017 to ensure universal health insurance coverage for the entire population with quality health services without facing financial hardship. [19] Beneficiaries receive health insurance plans through different schemes: public health insurance (either through the old health insurance system or the new UHI system) or private health insurance. [19] In 2020, the old health insurance organization (HIO) covered 56.985 million people (beneficiaries) with an expenditure per person of around EGP 280. [9] The new UHI system is in a pilot phase, currently covering six out of the 27 governorates (0.772 million beneficiaries in 2020) with EGP 1147 expenditure per person. It is planned that the new UHI system will be fully implemented across the country by 2030. [9]

Fig. 2 illustrates the share of current health expenditures per capita by financing scheme for the national average and Port Said (the pilot UHI governorate). As shown in Fig. 2, the implementation of the new UHI financing scheme in Port Said resulted in a substantial shift in the financing mix: the share of social health insurance expenditure increased from approximately 7% nationally to over 30% in Port Said, while household OOP payments fell from 59% nationally to 48% in Port Said. This pilot evidence is critical as it provides a real-world proof-of-concept that the UHI system can meaningfully reduce OOP burden and improve financial protection, supporting the scale-up rationale. **Supplementary Fig. S2** highlights the distribution of population coverage (in millions) by financing scheme in 2020, which shows that private health insurance covered 1.047 million people (with EGP 4285 spent per person), in contrast to the new UHI which covered 0.772 million (with 1147 spent per person).

**Fig. 2:**
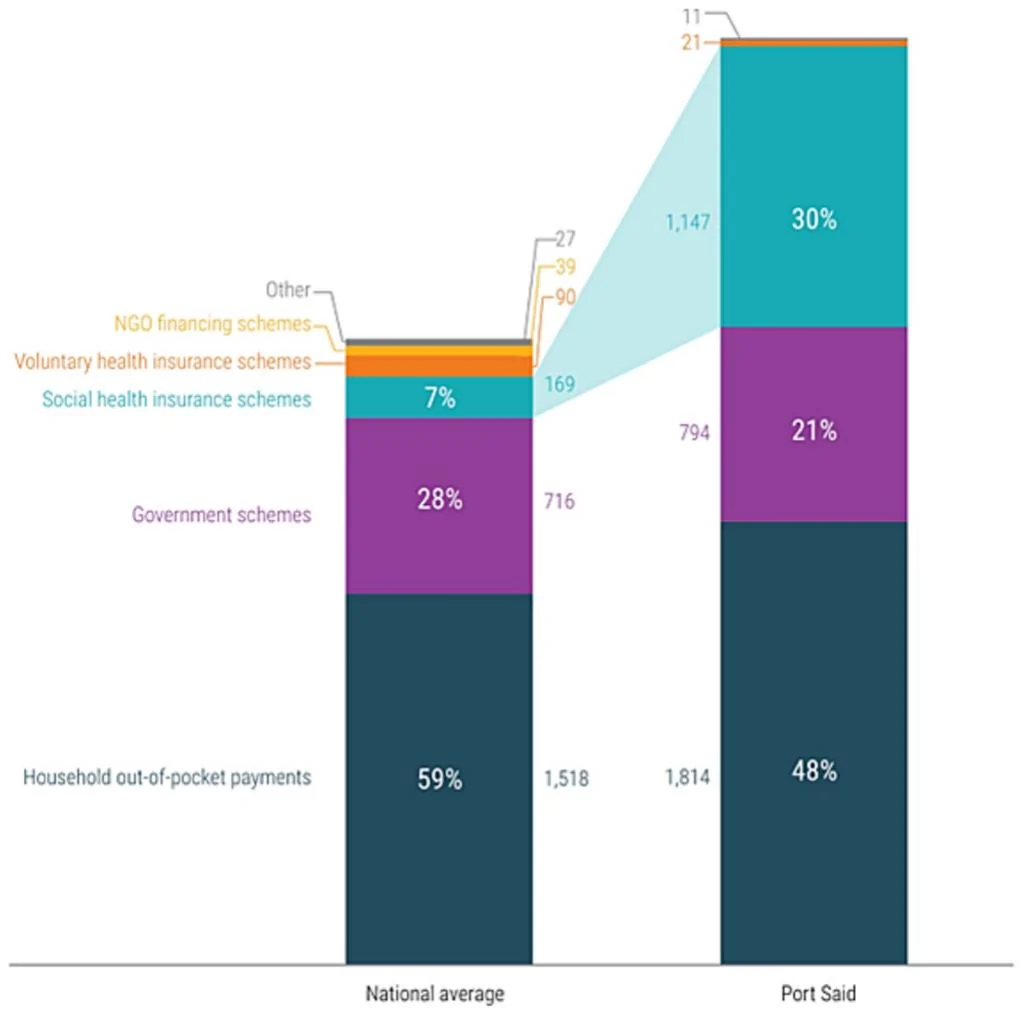
Share of current health expenditures per capita by financing scheme, national average, and Port Said (pilot phase for universal health coverage).

According to a paper published by Selwaness et al. (2019) about social protection and vulnerability in Egypt, the old tax-based health insurance system follows a proportional scheme for all citizens with exemption for the vulnerable populations, [20] while the new UHI covers all the vulnerable populations in the six governorates, with contributions from the Ministry of Finance for those who cannot afford to pay. [20] Despite this progress, there still remains some challenges related to population coverage for sub-groups of the population, [20] which were also highlighted during the focus group discussions as follows: “*although the poorest are better covered by the new UHI scheme, there remain coverage vulnerabilities for those in the informal economic sector such as the self-employed, for women particularly in rural areas, and for young people between the 20-24 age group*”.

Based on stakeholders' interviews, there are currently no risk equalization measures in place to ensure that financial resources are optimally allocated based on population needs rather than the ability to pay. One interviewee noted that “*there should be better processes for budgeting on the long term, optimal resource distribution and insurance coverage based on population needs, disease burden, and the consequential economic and social costs of diseases*”. Yet, there are currently no existing mechanisms in place to allocate funds based on population needs or disease burden. Furthermore, one focus group participant noted: “*We are still allocating based on historical spending patterns rather than epidemiological need — we lack the health information infrastructure to do otherwise*.” There was consensus among most stakeholders in the focus group discussions that cost projections and burden of disease estimates are conducted by the Universal Health Insurance Authority (UHIA) on a macro level based on the payer perspective only and not based on the healthcare provider or societal perspectives. On a micro level, there are currently undergoing initiatives by the Egyptian Drug Authority to conduct health technology assessment (HTA) of drug interventions, that may contribute to better allocation of resources based on cost-effectiveness estimates.

#### 3.2.3 Provider payment mechanisms

The financing scheme for paying healthcare providers differs based on the level of care (primary, secondary, and tertiary care) and the type of care delivery (inpatient, outpatient, intensive care, surgical care). This was explained by a key health insurance expert during the interviews noting that, “*Capitated payments for primary care services; fee-for-service payment for outpatient secondary/tertiary care and medical treatment (conservative/non-surgical); case-based payments for surgical interventions; and per-diem payments for intensive care and long-term conditions*”. Overall, the currently available payment models sustain the health system in the short term, as payment decisions are based on volume of services rather than value achieved.

Value-based payment (VBP) models have not yet been introduced in Egypt due to the absence of real-world data on efficiency indicators and health outcome measures at a national level, [21] therefore, it is difficult to link healthcare spending to health outcomes. 9., 10. However, experts' interviews indicated that transitioning to VBP should be prioritized in the medium- to long-term, contingent on the nationwide implementation of the universal health insurance and related health information systems. Shifting from fee-for-service to VBP would promote sustainability of health system financing by tackling excess health spending and growth, unnecessary administrative and clinical waste. 21., 22., 23. This finding directly addresses a policy recommendation by suggesting healthcare provider payment reform as a key lever for long-term sustainability.

Payments to health service providers are mainly attributed to retailers of medical goods for medicines, medical supplies, and equipment (32.9%), followed by hospitals (28.7%), and ambulatory healthcare providers (17.3%), 9., 10. constituting altogether 80% of the total money spent on healthcare providers, as shown in **Supplementary Fig. S3**. Such data is important to better understand the share of current health expenditure by each type of health service provider to be reflected on evidence-informed policy implications.

### 3.3 Resilience of Health system financing in Egypt during the COVID–19 pandemic

#### 3.3.1 Budgeting and crisis preparedness

During the COVID-19 pandemic, supplementary public funds were released from the Ministry of Finance to cover additional pandemic-related expenditures. Although there were no crisis preparedness contingency plans to deal with the COVID-19 pandemic, it was well-controlled and well-covered as the situation was not very severe in Egypt. In May 2021, Egypt's parliament approved a new bill establishing a medical emergency fund to combat emergency medical expenses with the aim of promoting resilience in health system financing. [24] The funds were allocated to patients receiving treatment at emergency and intensive care units, purchasing medicines, and providing accelerated healthcare services to reduce waiting lists during the COVID-19 pandemic. [24]

Stakeholder interviews provided insight into the mechanisms of this response as follows: *‘The government moved quickly to reallocate budget lines and release emergency transfers — there was political will but no formal playbook*.” This highlights both a strength (political responsiveness) and a weakness (absence of pre-planned financial risk management protocols) in Egypt's resilience capacity.

Although actuarial studies are conducted to estimate health spending in the short- and medium-term, there are no projections related to the risk of exposure to epidemics or pandemics. In addition, relative risk estimates are not currently measured for budget planning purposes. This represents a significant gap in financial resilience planning that must be addressed to prepare for future health shocks.

## 4 Discussion

This study provides valuable insights about the sustainability and resilience of health service delivery in Egypt based on the published literature, publicly available information on governmental websites, and insights from key stakeholders and experts in the field.

### 4.1 Strengths of this study

This study has several methodological strengths that enhance its scientific contribution. First, this is the first study to apply the PHSSR framework in Egypt, enabling a comprehensive and internationally comparable assessment of health system financing sustainability and resilience. The PHSSR framework's structured domains and methodology provided a unique and rigorous analytical scaffold that goes beyond the descriptive analyses available in prior Egyptian studies. Second, the multi-source triangulation design –combining literature, semi-structured stakeholder interviews, and expert focus groups– enriched the evidence base and allowed findings to be validated across data sources, strengthening the credibility of conclusions. Third, unlike purely quantitative analyses of health expenditure data, this mixed-methods approach captures the institutional dynamics, stakeholder perspectives, and contextual factors that underpin financing decisions. These dimensions are critical for policy translation and transferable to other lower-middle-income countries in the MENA region.

### 4.2 Key findings in context

Based on findings from the Egypt National Health Accounts 2019/2020 report, some indicators showed improved sustainability of health financing. [9] Egypt's health spending as a percentage of GDP increased between 2010 and 2020, while the OOP spending as a percentage of current health expenditures (CHE) decreased. However, it is important to address these positive indicators with acknowledgment of persistent structural challenges: OOP spending remains above 59% of CHE, the financing system is largely regressive, and coverage gaps persist for key vulnerable populations. The narrative of ‘improved sustainability’ should therefore be cautiously interpreted and understood as progress from a low baseline, not as evidence of an already sustainable system.

Key insights from the experts' interviews and focus groups demonstrated the resilience of the Egyptian health system financing during the COVID-19 pandemic, where supplementary public funds were released to cover the additional pandemic-related expenditures. Nevertheless, the resilience demonstrated during COVID-19, while notable, was reactive rather than proactive. The release of emergency funds represented political responsiveness rather than planned financial risk management. Moving forward, building genuine resilience requires institutionalizing crisis preparedness mechanisms including epidemic risk modelling, contingency budget lines, and pre-agreed activation protocols.

One of the aspects that negatively affects sustainability is the devaluation of the local currency (EGP) due to economic instability and high inflation rates. There are no existing mechanisms to allocate funds based on population needs or disease burden. Therefore, shifting from fee-for-service to VBP would promote sustainability of health system financing. 21., 22., 23.

### 4.3 Limitations

Despite the advantages of this study, several limitations exist that should be considered when interpreting the findings. First, there is a paucity of publicly available data and published literature on sustainability and resilience of health system financing in Egypt, which constrained the depth of the literature review component and the comprehensiveness of quantitative analysis. Second, the qualitative component relied on expert and stakeholder views collected in 2023, which may be subject to social desirability bias, where the participants may have presented the health system in a more favourable light given their professional affiliations. Efforts were made to mitigate this through purposive sampling of diverse stakeholders and iterative probing during interviews, but this limitation cannot be entirely eliminated.

Third, the findings reflect primarily the perspectives of urban-based professionals and policymakers. Rural populations, informal sector workers, and other marginalized groups are underrepresented. The absence of patient and citizen voices from the qualitative component is a particular limitation, as beneficiary perspectives are essential for a complete picture of financial protection. Nevertheless, it is a common norm in Egypt not to include the patients' voices due to lack of awareness about its importance. Fourth, regarding researcher positionality: the research team includes members affiliated with academic institutions in Egypt, WHO/EMRO, and the LSE. While this provides important contextual expertise, these affiliations may have influenced the framing of findings and recommendations based on respective knowledge and positioning. All interpretive decisions were made transparently and triangulated across team members to mitigate this risk. Fifth, the paucity of real-world national health outcome data makes it difficult to assess value-based reimbursement or link spending to outcomes. This data gap itself constitutes a finding of this study and is reflected in the recommendations.

### 4.4 Policy recommendations

We provided the following recommendations to promote the sustainability of health system financing, organized by timeframe and explicitly linked to the study findings that motivated them, to support evidence-informed policymaking.

**Table:** 

**Timeframe**	**Recommendation**	**Supporting finding**	**Responsible entity**
**Short-term** **(1–3 years)**	1. Develop financing schemes and strategic purchasing for services not covered by UHI, including preventive care, and immunization services. 2. Conduct micro-costing studies at the disease level based on the health provider perspective and societal perspective. 3. Develop financial risk management and crisis preparedness plans, including contingency budget lines for outbreak/pandemic response.	Coverage gaps for vulnerable populations (Section 3.2.2); Absence of actuarial pandemic risk models (Section 3.3).	MoHP, Ministry of Finance, UHIA
**Medium-term (3–5 years)**	4. Introduce risk equalization measures to allocate funds based on population needs, disease burden, and epidemiological data. 5. Adopt value-based payment (VBP) models for novel expensive drugs to link cost to health outcomes. 6. Implement an incentive system for efficient spending, linking costs of care to health outcomes.	No risk equalization mechanisms exist (Section 3.2.2); Fee-for-service limitations (Section 3.2.3).	UHIA, MoHP, health economists
**Long-term (5–10 years)**	7. Transition to value-based healthcare financing through nationwide health outcomes and costs-of-care measurement through health information systems. 8. Achieve full UHI rollout across all 27 governorates with progressive elimination of regressive OOP financing. 9. Make budgeting and spending projections based on disease burden and the economic benefit of health to society.	Regressive financing structure (Section 3.2.1); Port Said UHI pilot results (Section 3.2.2); Absence of outcome data (Section 4.3).	Government, MoHP, UHIA, World Bank

A key implementation challenge in the Egyptian context is the combination of economic instability, high inflation, and EGP devaluation, which constrain the fiscal space available for health investment. Additionally, the large informal economic sector creates structural barriers to contribution-based financing. The UHI system's success will depend on robust mechanisms to enrol and retain informal sector workers and address the ‘missing middle’ of coverage.

## 5 Conclusion

The health system in Egypt is currently financed through the old health insurance system, the newly implemented UHI system, private insurance, and OOP payments. Addressing the three research questions guiding this study: (1) the financing landscape is complex, with four concurrent schemes at different stages of implementation; (2) published data show modest but meaningful improvements in sustainability indicators between 2010 and 2020, though persistent structural challenges — including a regressive OOP-dominated financing structure and coverage gaps — require continued reform; (3) the health system demonstrated adaptive resilience during COVID-19 through emergency budget mobilization, though proactive preparedness mechanisms remain absent.

The successful pilot of the UHI system in Port Said provides real-world evidence for the feasibility of reducing OOP burden through universal coverage expansion. This study presents valuable insights about the current health system financing scheme in Egypt, evaluating the sustainability and resilience of financing the health system. The evidence-based recommendations provided in this study are organized by timeframe and linked to specific findings to facilitate uptake by policymakers in Egypt and transferable lessons for other lower-middle-income countries in the MENA region.

## CRediT authorship contribution statement

**Ranin Soliman:** Writing – original draft, Validation, Methodology, Formal analysis, Data curation. **Dan Gocke:** Writing – review & editing, Visualization, Validation, Methodology. **Elias Mossialos:** Writing – review & editing, Methodology, Conceptualization. **Hesham Dinana:** Writing – review & editing, Supervision, Project administration, Methodology, Funding acquisition, Conceptualization.

## Declaration of competing interest

The study was funded by AstraZeneca and Philips to conduct the work of the Partnership for Health System Sustainability and Resilience (PHSSR) project in Egypt. The funder had no role in the design, data collection, data analysis, reporting of this study, and the choice of the journal. The authors declare no other conflicts of interest.
