# Supplementary material for: Sustainability and resilience of health system financing in Egypt: a mixed qualitative methods study for evidence-informed policymaking
Source: Health Policy Open. 2026 Oct 28;11:100194. doi: 10.1016/j.hpopen.2026.100194 (PMC13755857; doi:10.1016/j.hpopen.2026.100194)
Supplement: Supplementary file 1 [file mmc1.docx]

**Supplementary Materials**

**Sustainability and Resilience of Health System Financing in Egypt: A Mixed Qualitative Methods Study for Evidence-informed Policymaking**

**Supplementary Table S1.** Interview guide based on PHSSR health system financing areas

**Supplementary Figure S1.** Current health expenditure as a percentage GDP and per capita, 2001–2021

**Supplementary Figure S2**. Distribution of health expenditures by financing scheme, coverage, and per person

**Supplementary Figure S3.** Paying health service providers– share of current health expenditure by type of provider

**Supplementary Table S1. Interview Guide based on PHSSR health system financing areas**

| **Areas/topics** | **Guiding questions** |
| --- | --- |
| 1. **Budgeting and crisis preparedness** | • Describe what processes are in place to diagnose and monitor the fiscal sustainability of the health sector. Are there any spending projections, and if so, what period do they cover?  • Are projections in the burden of disease, the consequential economic and social costs of disease, and the broader economic benefits of health to society undertaken to inform health budget planning and funding decisions?  • Are projections undertaken related to the risk of exposure to epidemics/pandemics, and do changes in the relative risk affect budget planning?  • Are financial risk-management plans in place to build up reserves or ensure additional public funds can be released for use during crises?  • Have provisions for contingency financing been reformed in light of the experience of the COVID-19 pandemic? |
| 2. **Revenue generation and spending** | • Who is responsible for raising revenue, setting contributions, paying providers, and capital expenditure? How are financing decisions delegated to regions and localities? How are investments in the health system valued relative to other areas of public spending?  • Does the country face a narrowing tax base due to an aging population, and are plans underway to address this issue?  • Is healthcare financing overall progressive or regressive? Are there any specific areas of regressivity?  • If the system is funded by social health insurance, do the insurance funds run a deficit or a surplus? Has the proportion of funding from general taxation versus payroll taxes (employer and employee contributions) changed in the last 10 years?  • Are there mechanisms for bailing out public providers and/or social insurance funds that run into deficits? Have bailouts been necessary in the past 10 years?  • Was there a mechanism to make additional funding available to support the response and management as well as maintain essential services at the onset of the COVID-19 pandemic? Was this used effectively during the pandemic, and did public health bodies have sufficient funds during the crisis?  • Had an excessive focus on efficiency gains and cost-containment pre-COVID-19 undermined the system’s resilience when the crisis hit? |
| 3. **Coverage and resource allocation** | • Is the distribution of spending across social care; primary, secondary, and tertiary care; and public health sufficient? Has this changed since the COVID-19 pandemic?  • Are there risk equalization measures to ensure that resources are allocated (geographically or between insurance funds) based on population needs, rather than ability to pay?  • What are the largest gaps in coverage, in terms of who is covered, what is covered, and what share of total costs are covered? Do unemployed or self-employed people as well as vulnerable groups have access to health coverage? What is the rate of catastrophic healthcare expenditure (defined as out-of-pocket expenditure exceeding 10% of household consumption)? Paying providers  • Does the way primary, secondary, and tertiary care providers are typically paid (e.g., through budget payments, fee-for-service payments, capitated payments, or case-based payments) promote sustainability and resilience in the health system?  • Have value-based payment models been introduced in primary, secondary, and tertiary care? |
| 4. **Key trends in health system financing** | • How has the share of healthcare spending as a proportion of gross domestic product changed in the last 10 years, or since the onset of the COVID-19 pandemic?  • How has the share of public and private sources of funding (social health insurance, taxation, private insurance, or out-of-pocket spending) changed in the last 10 years or since the onset of the COVID-19 pandemic?  • How has the proportion of people over age 65 changed over the last 10 years?  • How has the rate of participation in the formal labor force changed in the last 10 years and since the onset of the COVID-19 pandemic? |

**Supplementary Figure S1. Current health expenditure as a percentage GDP and per capita, 2001–2021**

**Note:** GDP = Gross domestic Product; **Source:** WHO Global Health Expenditure Database (2024)

**Supplementary Figure S2**. **Distribution of population coverage (in millions) and expenditure pr person (EGP), by financing scheme**

**Source:** WHO Global Health Expenditure Database (2024)

**Supplementary Figure S3. Paying health service providers– share of current health expenditure by type of provider**

**Source:** WHO Global Health Expenditure Database (2024)
